# Odorant-binding proteins in canine anal sac glands indicate an evolutionarily conserved role in mammalian chemical communication

**DOI:** 10.1186/s12862-021-01910-w

**Published:** 2021-09-26

**Authors:** Sunita Janssenswillen, Kim Roelants, Sebastien Carpentier, Hilde de Rooster, Mieke Metzemaekers, Bram Vanschoenwinkel, Paul Proost, Franky Bossuyt

**Affiliations:** 1 Amphibian Evolution Lab, Biology Department, Vrije Universiteit Brussel, Pleinlaan 2, 1050 Brussels, Belgium; 2 Proteomics Core - SyBioMa, Katholieke Universiteit Leuven, Herestraat 49 - 03.313, 3000 Leuven, Belgium; 3 Small Animal Department, Faculty of Veterinary Medicine, Ghent University, Salisburylaan 133, 9820 Merelbeke, Belgium; 4 Rega Institute, Molecular Immunology, Katholieke Universiteit Leuven, Herestraat 49 - Bus1042, 3000 Leuven, Belgium; 5 Community Ecology Lab, Biology Department, Vrije Universiteit Brussel, Pleinlaan 2, 1050 Brussels, Belgium; 6 Center for Environmental Management, University of the Free State, Bloemfontein, 9030 South Africa

**Keywords:** Odorant binding proteins, Chemical communication, Anal sac glands, Placental mammals, Carnivores, Dogs

## Abstract

**Background:**

Chemical communication is an important aspect of the behavioural ecology of a wide range of mammals. In dogs and other carnivores, anal sac glands are thought to convey information to conspecifics by secreting a pallet of small volatile molecules produced by symbiotic bacteria. Because these glands are unique to carnivores, it is unclear how their secretions relate to those of other placental mammals that make use of different tissues and secretions for chemical communication. Here we analyse the anal sac glands of domestic dogs to verify the secretion of proteins and infer their evolutionary relationship to those involved in the chemical communication of non-carnivoran mammals.

**Results:**

Proteomic analysis of anal sac gland secretions of 17 dogs revealed the consistently abundant presence of three related proteins. Homology searches against online databases indicate that these proteins are evolutionary related to ‘odorant binding proteins’ (OBPs) found in a wide range of mammalian secretions and known to contribute to chemical communication. Screening of the dog’s genome sequence show that the newly discovered OBPs are encoded by a single cluster of three genes in the pseudoautosomal region of the X-chromosome. Comparative genomic screening indicates that the same locus is shared by a wide range of placental mammals and that it originated at least before the radiation of extant placental orders. Phylogenetic analyses suggest a dynamic evolution of gene duplication and loss, resulting in large gene clusters in some placental taxa and recurrent loss of this locus in others. The homology of OBPs in canid anal sac glands and those found in other mammalian secretions implies that these proteins maintained a function in chemical communication throughout mammalian evolutionary history by multiple shifts in expression between secretory tissues involved in signal release and nasal mucosa involved in signal reception.

**Conclusions:**

Our study elucidates a poorly understood part of the biology of a species that lives in close association with humans. In addition, it shows that the protein repertoire underlying chemical communication in mammals is more evolutionarily stable than the variation of involved glands and tissues would suggest.

**Supplementary Information:**

The online version contains supplementary material available at 10.1186/s12862-021-01910-w.

## Background

Carnivores use anal sac gland secretions (ASGS) to communicate by smell, whereby each individual is believed to produce a distinct chemical profile, composed of a unique blend of scent molecules [1]. The exchange of chemical profiles between individuals of the same species can be a key determinant of their subsequent social interaction. Behavioural experiments have shown that ASGSs are used among conspecifics for territorial marking (wolf, hyena) [2–4], and for identification of gender (ferret, brown bear) [5, 6], individuality (domestic cat, honey badger, hyena, mongoose) [7–10], hierarchic status (hyena) [8, 11], familiarity (ferret, hyena) [5, 11], and kin recognition (meerkat) [12].

**Fig. 1:**
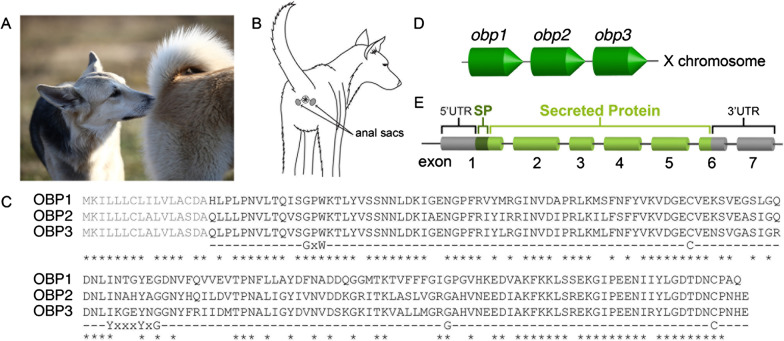
Odorant-binding proteins (OBP) in anal sac gland secretions of dogs. **A** Picture of dog behaviour that shows the investigation of anal zones. **B** Canine anal sacs are situated at both sides of the anus. **C** Sequence alignment of OBP isoforms of canine ASGS. The signal peptide and the functional protein are marked in grey and black, respectively. Stars show identical amino acids in all proteins. Signature residues and motifs that are conserved among most mammalian lipocalins are included below the amino acid sequences. **D** *Obp* gene organisation in the dog genome. The *obp* gene cluster is situated on the X chromosome and obtains three genes with nucleotide positions of *obp1*: 2,893,438–2,899,738, *obp2*: 2,905,438–2,911,062, and *obp3*: 2,918,198–2,923,699. **E** *Obp* gene structure: every dog *obp* gene contains seven exons of which exons 1 to 6 span the coding region. UTR = untranslated regions at the 5ʹ and 3ʹ ends; SP = signal peptide

Dogs (*Canis lupus familiaris*), of all carnivores, arguably live in closest association with humans and the importance of anal scent assessment in their social behaviour is known well beyond the scientific community. Indeed, when dogs meet, their sniffing behaviour reveals an obvious focus on the perianal zone that contains the anal sac glands (Fig. 1A, B) [13, 14]. Such scent assessment not only takes place during a first encounter but also on a daily basis among pack members. ASGSs may also be used by dogs for scent marking when released together with faeces, to advertise their presence and/or territory, as shown in wolves [2, 3]. Finally, during fear-induced responses, the entire content inside the anal sacs can be released as a spray, resulting in an intense foul odour and suggesting a common alarm or defence scent signal [13, 15, 16]. Despite many indications of the multifunctionality of anal sac glands in dogs, investigation of their molecular contents has remained limited to early gas chromatography studies of volatile compounds [17, 18].

Anal sac glands are modified sweat and sebaceous apocrine glands that are a unique trait of carnivores [1]. The sacs are often hypothesized to be fermentation chambers in which small volatile metabolites produced by gland-inhabiting bacteria are stored and secreted to be used as scent signals [19–25]. Consistent with this hypothesis (and because volatile molecules are most effective in creating a scent) most studies on carnivores have focused on the identification of small volatile molecules [5, 8, 10, 18, 21, 26–30]. However, proteins endogenously produced by the animal (and thus encoded by genes in its genome) could be similarly important for chemical communication. Indeed, endogenously produced proteins have been shown to play an important role in chemical signalling in noncarnivoran mammals like rodents [31–37], primates [38] and pigs [39, 40]. Despite early predictions of the presence of proteins in carnivoran anal sac glands [41], only one protein has ever been identified from the ASGS of a carnivore [42].

To investigate the importance of proteins in carnivore chemical communication, we conducted a comprehensive proteomic study of the ASGS of dogs using published genome sequences as a reference. To reconstruct the evolutionary history of the newly found proteins, we used comparative genomics and phylogenetic analyses on related genes of a wide taxonomic range of mammals. Our findings, besides providing new insights in the functioning of canine scent glands, further elucidate the evolution of chemical signalling in carnivores and, by extension, in mammals.

## Results

### Identification of proteins in canine ASGS

We sampled the anal sac secretion of 17 dogs of different ages, including six males and 11 females (Additional file 1). Four females were sampled twice, once during anoestrus and once in oestrus during their fertility peak. Bicinchoninic acid (BCA) protein assays indicated variable protein concentration across canine anal sac samples and animals, ranging between 5.44 and 868.68 mg/ml (21 samples from 17 individuals; Additional file 1). Despite this variation in concentration, SDS-PAGE and RP-HPLC indicated a similar protein composition across all individuals. For three samples, the highest chromatogram peak was analysed using a combination of Edman sequencing and electrospray ionization mass spectrometry. It represented a protein of 17,468.28 Da with the following 24 N-terminal amino acids: HLPLPNVLTQIxGPxKTLYVSSNN. A BLAST search against the Uniprot database [43] identified this sequence as part of an Odorant Binding Protein (OBP), a subclass of structurally related proteins within the lipocalin family that can bind a wide range of volatile molecules [44, 45].

To obtain a general overview of proteins secreted by dog anal sacs, we conducted liquid-chromatography–tandem mass spectrometry (LC–MS/MS) on all 21 samples. This analysis showed the existence of not one, but three OBP isoforms (Fig. 1C), all of which were present in all samples, albeit at varying abundances (Additional files 2 and 3). No apparent differences were observed between age classes, genders, or oestrus states, but OBP abundances were invariably higher in mixed breeds (stray dogs) than in crossbreds and purebreds (Additional file 2).

Besides OBP, LC–MS/MS revealed a large diversity of secretory proteins, many of which have previously been shown to play a role in the mammalian immune system. In total, peptide fragments of 57 unique proteins at 0.01 false discovery rate (FDR; Additional file 3) were sequenced. Four of these represent antimicrobial proteins: lactotransferrin (LTF), cathelicidin, prolactin-induced protein (PIP) and C-type lysozyme. In various mammals, each of these proteins has been shown to kill bacteria or control their growth through different mechanisms [46–49]. We also found six immunoproteins: polymeric immunoglobulin receptor (PIGR), joining chain of multimeric IgA and IgM (JCHAIN), Immunoglobulin Heavy Chain-like (IGH), Zinc-Alpha-2-glycoprotein (AZGP1), and two Ig domain-like containing proteins. In humans and other mammals these proteins are involved in antigen processing and antigen presentation as part of the humoral immune system [50–53]. In addition, several types of protease inhibitors (WAP-type, KAZAL-type protease inhibitors, serpins, cystatins and alpha-2 macroglobulins) were identified in the canine anal sac content. The exact function of these proteins in ASGSs is unclear but by controlling the activity of proteases, they may contribute to a wide range of biological processes [54]. Serum Albumin (SA) represents a final consistent constituent of ASGS. Besides being a transport protein in blood, SA has also been described as part of the pheromone signalling complex in Asian elephants (*Elephas maximus*) [55].

### Structural features of odorant binding proteins

Canine OBPs are small (173 or 174 amino acids) extracellular proteins that share a sequence similarity of 67% (Fig. 1C). Tertiary structure prediction using the online tool PHYRE2 [56] confirmed that all three OBPs form an eight-stranded antiparallel beta barrel, a structure that has been previously determined for OBP3 (identified as the allergen Can f 4 in dog dander) [57] and is shared among members of the lipocalin family [58]. They can reversibly bind a broad range of small organic compounds in their hydrophobic pocket structures [44, 45, 59, 60]. As part of the lipocalin family, OBPs contain a GxW motif (positions 29–31), a glycine residue (position 136), and two cysteines (positions 78 and 170; forming a disulphide bridge), as characteristic sequence signatures shared among most lipocalins [45]. An additional lipocalin-specific motif, YxxxYxG (positions 93–99) was found to be only partially conserved in canine OBPs (Fig. 1C).

### Canine anal sac gland OBPs are encoded by a gene cluster on the X-chromosome

Screening of the dog genome (UCSC, NCBI and Ensembl genome browsers) revealed that the anal sac OBP isoforms are encoded by three different genes organised in a single cluster on the X chromosome (Fig. 1D). None of the isoforms showed evidence of alternative splicing or the combined transcription of exons of multiple genes (yielding chimeric proteins), as confirmed by our LC–MS/MS protein data (not shown). For *obp3*, encoding the dander allergen Can f 4, a transcript was previously cloned [61]. Mapping of this transcript and the protein sequences inferred here indicated that the three genes share the same structure composed of seven exons (Fig. 1E) with coding regions spanning exons 1 through 6. Peptide abundance data inferred from our LC–MS/MS analysis shows that the first 16 amino-acids encoded by these regions are not included in the secreted proteins, indicating the posttranslational excision of a conserved N-terminal fragment. This fragment (MKILLLCLILVLA^C^_S_DA) is confirmed to be a signal peptide, characterising secretory proteins.

### Conserved synteny of the *obp* gene repertoire in placental mammals

**Fig. 2:**
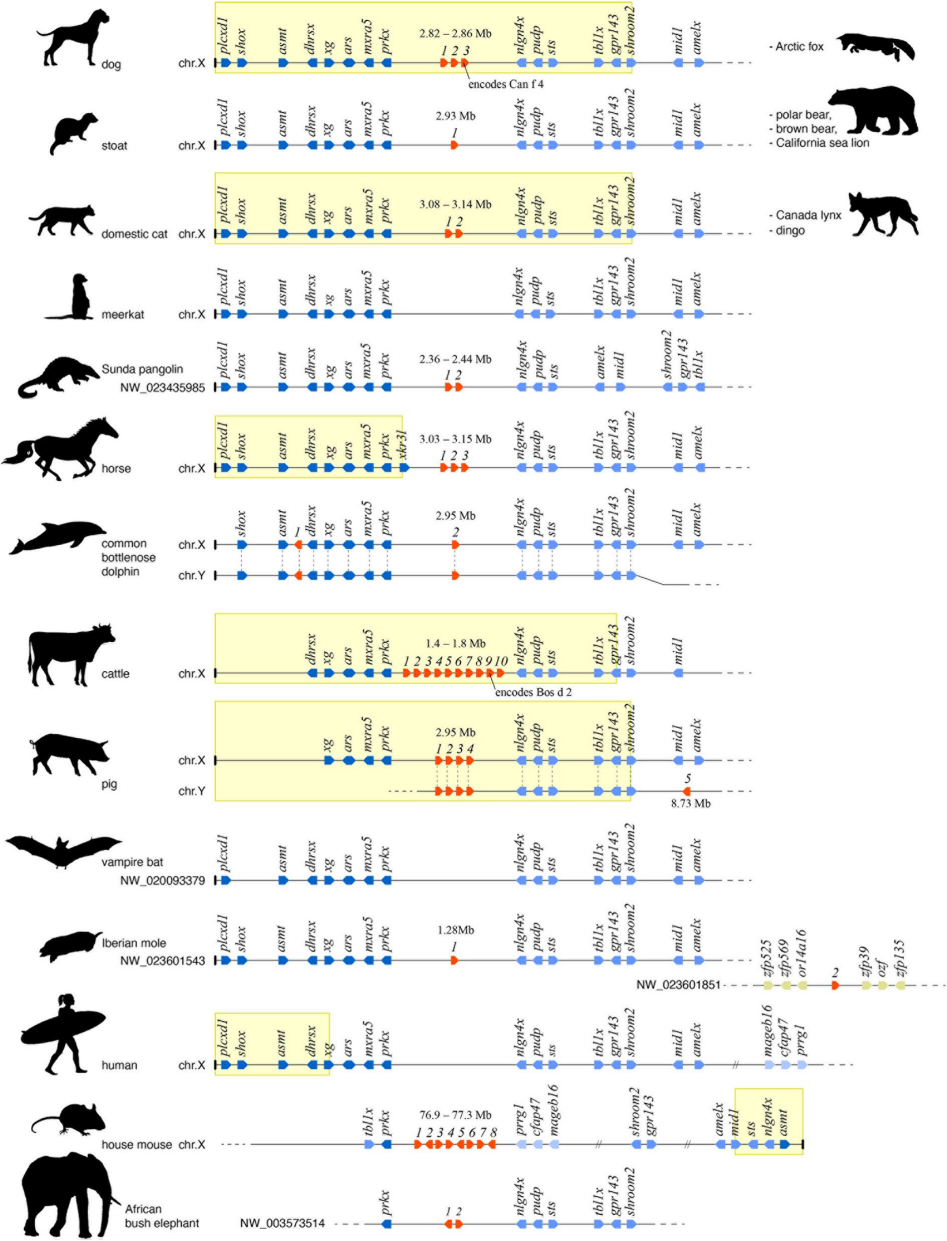
Variation in size and genomic organisation of *obp* gene repertoires in placental mammals. Each line represents a schematic map of the genomic organisation of *obp* genes (in red) and flanking genes (in blue) for the species and chromosome/scaffold indicated on the left. Species sharing the same organisation are listed on the right. Individual genes are depicted as arrows indicating their orientation on the chromosome. To visualise the shared synteny, orthologous genes are vertically aligned across species when feasible. Shades of blue of flanking genes indicate whether they typically lie distal (dark blue), proximal (medium blue) or at a large distance (light blue) from the *obp* locus. Genes on a different genome scaffold (in Iberian mole) are coloured ochre. Numbers above *obp* genes indicate their distance (in Mb) from the nearest chromosome end. Known chromosome ends are indicated by a vertical cap typically on the left of a map. Yellow windows in delineate known pseudoautosomal regions

To investigate the evolutionary history of the dog’s *obp* repertoire, we used the sequences of the newly discovered dog proteins to screen 98 genomes, including those of 19 other carnivores as well as representatives of the major mammalian lineages. Homologous genes sharing synteny with the dog’s *obp* genes (as evidenced by the same order of flanking genes) were found in 35 placental species representing a wide phylogenetic range (Additional file 4). In 15 of those, *obp* homologues were confirmed to be situated on the X-chromosome (Fig. 2). In the majority of these species, the *obp* locus is situated in a relatively distal position on the X-chromosome, at distances below 4 Mb from one of the chromosome’s ends. Such distal position is likely within the pseudoautosomal region (PAR) of the X-chromosome, a short region that maintains high sequence similarity with a corresponding region on the Y-chromosome, allowing recombination during meiosis in males [62]. Based on inferred pseudoautosomal boundaries in the genes *grp143* and *shroom2* [63–65], our genome mapping confirms the position of the *obp* locus within the PAR for cattle (*Bos taurus*), pig (*Sus scrofa*), cat (*Felis catus*) and dog (Fig. 2). In horse (*Equus caballus*) however, the *obp* gene cluster lies outside the PAR due the presence of a more distal boundary, within a horse-specific *xkrp3*-like gene [62, 66]. In house mouse (*Mus musculus*), the *obp* gene cluster lies in a far more central position of the X-chromosome, at over 75 Mb distance from a very short and modified PAR [67]. The mapping of *obp* genes within the PAR of the X-chromosome implies that identical copies or alleles are very likely present in the corresponding PAR of the Y-chromosome. Indeed, BLAST screening revealed the presence of identical *obp* gene copies on the Y-chromosome in pig and common bottlenose dolphin (*Tursiops truncatus*), two of only few species for which separate sequence data for the Y-chromosome are available. In pig, one gene copy was additionally found in the nonautosomal region of the Y-chromosome, at approximately 8.73 Mb from the chromosome’s end (Fig. 2). If correct, this observation implies the origin of a male-specific *obp* gene copy. Screening of five marsupial and two monotreme genomes reveals the strong synteny of genes that flank *obp* genes in placental mammals. However, in all cases, this synteny is restricted to autosomal chromosomes, suggesting that their position on the X-chromosome is unique to placental mammals. Similarly, no *obp* genes were found in any marsupial or monotreme genome.

### Dynamic evolution of the *obp* gene repertoire in placental mammals

Placental mammals show substantial variation in the number of *obp* genes, suggesting differential rates of gene diversification across taxa. Among carnivores, canids (dogs and foxes) share the largest number (three) of *obp* genes. Remarkably however, the dingo (*Canis lupus dingo*, Australia’s feral dog) only has two *obp* genes despite its very recent (and incomplete) divergence from domesticated dogs [68]. The majority of carnivores as well as other placental species share one or two *obp* genes. Absence of *obp* genes was observed for meerkat (*Suricata suricatta*), all bats (19 genomes screened), Chinese treeshrew (*Tupaia chinensis*), Sunda flying lemur (*Galeopterus variegatus*) and all primates (29 genomes screened). For some species (most eulipotyphlans and all lagomorphs), the presence, number and organisation of *obp* genes could not be established due to the fragmentary nature of the available draft genomes. The largest *obp* gene repertoires are found in rodents, pig and pecorans (horned mammals including cattle). While this repertoire has been well characterised in house mouse [69–73] and golden hamster [74–76], only few *obp* genes of pig and cattle encode previously identified proteins [77–79]. In several species, identified *obp* homologues are probably pseudogenes (Additional file 4). All *obp* homologues found in pinniped species (walrus, sea lions and seals) share premature stop codons, while in cetaceans (whales and dolphins), both *obp* homologues are apparently missing exons. In other species, additional exon and gene fragments were found in the vicinity of identified *obp* genes, indicating duplication and/or loss of incomplete genes.

**Fig. 3:**
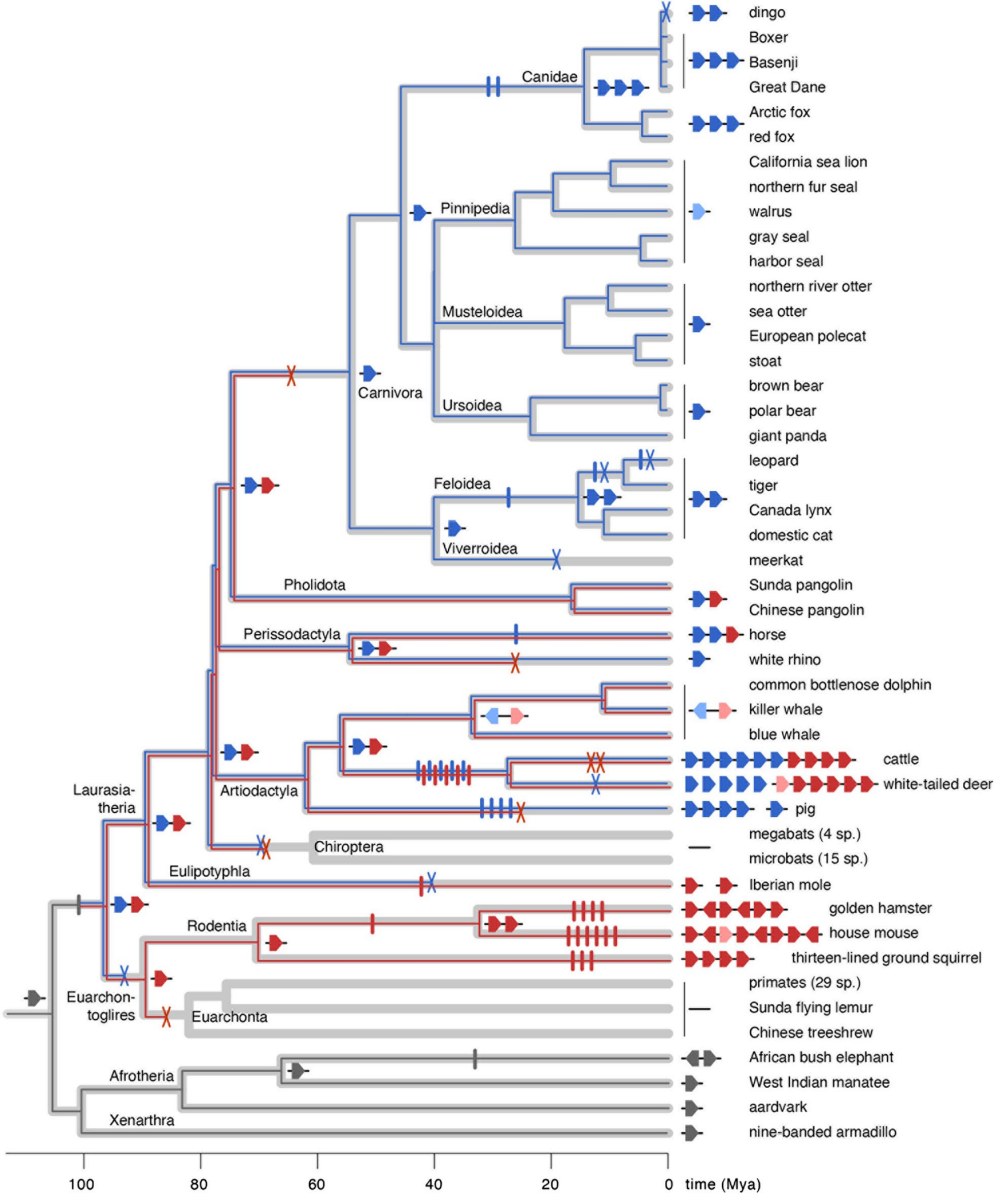
Evolutionary history of the *obp* gene repertoire in placental mammals. The taxonomic diversification of placental mammals is depicted here as a timetree (thick light grey branches) with divergence times inferred from ref. 84 (see “Methods”). *Obp* gene lineages are drawn as thin branches superimposed on the timetree with gene duplication events and gene losses along branches depicted as vertical bars (I) and crosses (X), respectively. Ancestral and extant gene repertoires are schematically shown at key internal nodes and terminal nodes respectively. Red and blue branches and genes represent two paralogous gene lineages that descended from a gene duplication inferred at the base of the placental radiation, in the ancestor of Euarchontoglires and Laurasiatheria. Probable pseudogenes are coloured in lighter shades. The reconstruction was obtained by reconciling an estimated gene tree with the mammalian timetree while inferring the lowest possible number of gene duplication events and losses. As a gene tree, we used a consensus phylogram from Bayesian and nonparametric bootstrapping analyses in which weakly supported branches (posterior probabilities < 0.9 and bootstrap percentages < 70%) were allowed to be rearranged if this reduced the number of inferred gene duplication events and losses. An alternative reconstruction in which such rearrangements were not allowed is shown in Additional file 6

Phylogenetic analyses of 102 sequences retrieved from three dog breeds, dingo and 36 other placental species further elucidate the evolutionary history of the *obp* gene repertoire (Additional files 4 and 5). Because high sequence divergence between *obp* and other lipocalins complicates sequence alignment and may lead to spurious rooting and poor branch support, we conducted phylogenetic analyses without outgroup sequences. The resulting unrooted *obp* gene tree is relatively well resolved, with high Bayesian posterior probabilities (BBP > 0.95) and nonparametric bootstrap percentages (NPBS > 75%) for major clades grouping *obp* sequences from the same mammalian taxa, like Carnivora, Pecora, Rodentia and Afrotheria. To reconstruct *obp* gene diversification throughout the placental mammalian evolutionary history, we performed a gene-tree/species-tree reconciliation (GTSTR) analysis, which superimposed the inferred gene tree on the phylogeny of placental mammals [81–84] while minimizing the inferred number of gene duplication events and losses. A GTSTR analysis using a fixed gene tree (prioritising confidence in the gene tree including weakly supported branches over minimising the number of events) reconstructed 42 gene duplication events and 30 gene losses (Additional file 6). Instead, when rearrangement of weakly supported branches (BPP < 0.9 and NPBS < 70%) was allowed in favour of an improved reconciliation, a far more parsimonious reconstruction was obtained, with 38 duplications events and 15 losses (Fig. 3). This difference reflects an effect of phylogenetic uncertainty in the gene tree and is mostly restricted to basal branches in the mammalian tree (Additional file 6). Despite their differences, both reconciliations indicate that *obp* gene duplication started during the early placental radiation, creating at least two basal paralogues before the divergence between Laurasiatheria (including carnivores, artiodactyls and relatives) and Euarchontoglires (including rodents, primates and relatives). One *obp* paralogue (indicated by blue lineages in Fig. 3) was subsequently lost in the ancestors of Euarchontoglires (incl. rodents, primates and relatives) and Chiroptera (bats) and along the branches leading to meerkat and Iberian mole (*Talpa occidentalis*). However, the same paralogue duplicated further in canids, felids, horse, pig and pecorans. A second paralogue (indicated by red lineages in Fig. 3) was lost in the ancestor of Euarchonta (primates and relatives), bats, carnivores, white rhinoceros (*Ceratotherium simum*) and pig but diversified in rodents and pecorans. The shared presence of three *obp* paralogues in dogs and foxes is explained by two gene duplication along the canid stem lineage. Within the dingo, the lost gene turns out to be *obp3* (Can f 4).

**Fig. 4:**
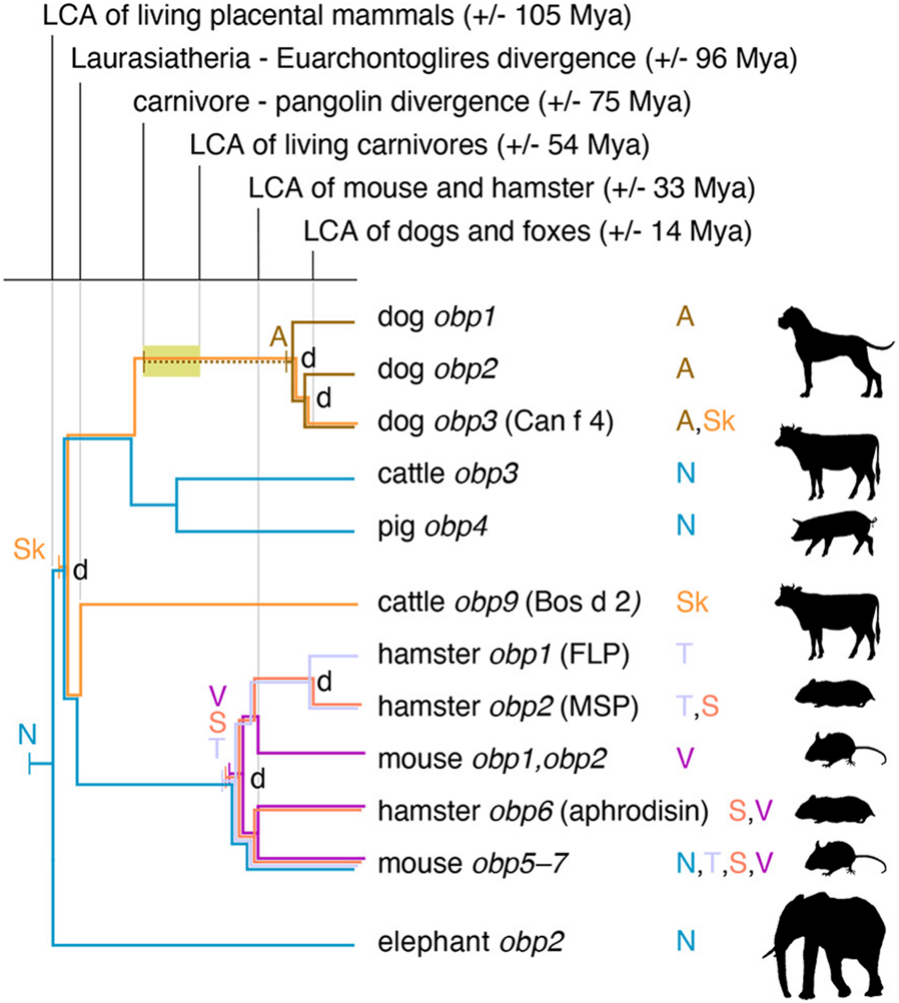
Tentative reconstruction of the evolution of new OBP secretion sites. Information on expression sites of extant *obp* genes is inferred from the present study (dog *obp*) and previous proteomic studies of nasal mucosa (N), saliva (S), skin (Sk), tears (T) and vaginal secretion (V; Additional file 7). The depicted tree is based on the previously inferred gene tree pruned to include only genes for which expression information are available and modified to fit placental mammal divergence times retrieved from Ref. [84]. Nodes representing gene duplication events are labelled with ‘d’. For each expression site, the history is traced back (coloured branches) to a single origin (a labelled vertical bar). Origins were mapped on the tree by ancestral state reconstruction using the parsimony principle (see “Methods”). Uncertainty in the origin of OBP secretion in anal sac glands is indicated by a dashed line. The time window in which anal sac glands most likely originated is shown as a yellow window

Absence of tissue expression data for the majority of presently identified *obp* genes precludes a detailed reconstruction of the evolutionary changes in OBP secretion. However, our finding of OBP in canid anal sac glands combined with previously published proteomic data on OBP in elephant, rodents, pigs and cattle (Additional file 7) implies the expansion of expression sites across multiple mammalian taxa to include their presence in nasal mucosa, skin, saliva, tears, vaginal secretion and anal sac gland secretion. Noteworthy, these sites the evolution of functions at the receiving end of chemical communication (nasal mucosa) as well as at the signalling end (all others). Mapping of these secretion data on a pruned version of the inferred *obp* gene tree using the parsimony principle yields a first tentative image of OBP expression evolution (Fig. 4). The most parsimonious reconstruction (assuming a single ancestral origin for each observed secretion site and not counting losses) suggests that nasal mucosa was the original expression site in the last common placental ancestor, with skin expression originating no later than the basal *obp* gene duplication during basal placental diversification (see also Fig. 3). The origin of lacrimal, salivary and vaginal secretion in rodents must have happened no later than the last ancestor of golden hamster and house mouse. Finally, as anal sac glands are a carnivore synapomorphy, they must have originated along the stem branch of Carnivora, after its divergence from Pholidota (pangolins) and before the last common ancestor of all carnivores (ochre time window in Fig. 4). Consequently, OPB secretion in these glands must have similarly originated after the carnivore–pangolin divergence and before the duplication events that created three canid paralogues in an ancestor of dogs and foxes.

## Discussion

Research of various mammal species has demonstrated that OBP proteins are involved in two key phases of chemical communication. In vaginal secretions, saliva, tears and urine of rodents, they are an essential part of signalling complexes that are exchanged during sexual and other social interactions [32, 35, 59, 69, 85–89]. In nasal mucosa of elephants, rodents, and some ungulates (pig, cow, goat, sheep), OBP proteins assist with the perception of molecules inhaled from the environment [77, 78, 90–94]. The function of OBP in other mammals and the role of anal sac glands in carnivore scent signalling together provide a compelling indication that canine OBP proteins contribute to conspecific chemical communication. Fuelled by the fermentation chamber hypothesis, anal sac research has predominantly pursued the identification of microbiomes and associated volatiles [5, 8, 10, 18, 21, 26–30]. However, our finding of OBP proteins as major components in ASGSs indicates that endogenously produced proteins are a crucial part of this signalling system as well. Since canids share anal sac glands with other carnivores (felids, hyenas, mongooses, bears and musteloids), the involvement of OBP in anal scent communication may be more widespread than currently known. Only one protein-related study of carnivore anal sac glands has been performed. In the domestic cat, the protein Fel d 1, a secretoglobin described as a human allergen and unrelated to lipocalins, was identified [42]. However, as this study targeted this protein using immunochemistry, no broader information on protein content of feline ASGSs is known.

OBP proteins may perform a range of biological functions in ASGSs. First, these proteins could transport and store odorants (the actual scent signals) by the formation of protein–ligand complexes [45]. Second, OBP proteins could extend the preservation of a signal in faecal scent marks, by delaying the release of their ligands into the environment [95, 96]. Third, selective binding of specific ligands could control the information that is presented to the receiving animal. This observation was made for SAL1 in pigs [40] and aphrodisin in hamsters [59]. Fourth, OBP proteins could act as a trigger to release volatile ligands during investigation by another animal. Dogs tend to press their nose against another individual’s anal zone or scent mark, sometimes even licking it. This contact could change the physicochemical environment of the transport protein–ligand complexes, causing their dissociation. Such mechanism has been postulated for elephants [55]: pre-ovulating females release urine marks which contain complexes of SA proteins and volatile sex pheromones. The trunk of a male inspecting a female urine mark changes the acidic environment while touching the urine, which triggers the release of the SA-pheromone complex, creating a burst of pheromone scent. Finally, OBP proteins could act as signals themselves. This function was previously shown in mice for major urinary proteins (MUPs), distantly related lipocalins that are involved in sexual attraction and individual recognition [34, 37].

Scent in dogs is thought to convey information regarding age, gender, heat cycle, social status, health and fitness [97, 98]. One question that arises from our findings is whether OBP variation across dog individuals reflects such information. Although we investigated only a limited number of individuals, OBP abundances seemed consistently higher in mixed breeds than in crossbreds and purebreds (Additional files 2 and 3). A possible explanation for this observation could be different social environments in which the dogs were born. Purebred dogs typically descend from pedigrees characterised by limited social structure and non-competitive mate selection controlled by humans. In contrast, all mixed-bred dogs of the current study were captured as stray dogs by animal shelter organisations on the streets of Romanian and Spanish towns, where they were part of large packs. Such packs are characterised by a well-developed hierarchic structure and mate selection controlled by highly competitive social interaction [99]. In such a complex social environment, a well-functioning communication gland is essential. An expanded comparative study including additional breeds and integrating observations of mate choice in feral conditions could substantiate this hypothesis.

Besides a general role in social interaction, OBPs of several mammalian taxa have been shown to underlie chemical communication related to sexual reproduction. In this perspective, the position of *obp* genes on the X-chromosome enables a possible genetic mechanism for the evolution of new sex-specific signals. Our genomic analyses show that *obp* genes of dog, cat, cattle and pig lie within the pseudoautosomal region (PAR). Similar distal positions in other taxa (Fig. 2) suggest that this pattern may be common in placental mammals. The PAR is a segment of typically less then 10 Mb that shares 96–100% sequence identity with a corresponding segment on the Y-chromosome [62]. Consequently, *obp* genes in many species may have identical copies or closely related alleles on the Y-chromosome. In house mouse and horse however, the *obp* loci lie outside the PAR, due to their relocation to a far more proximal locus (mouse), or through the origin of a new pseudoautosomal boundary slightly more distal than the *obp* gene cluster (horse [66]; Fig. 2). Either way, an X-specific position may preclude recombination during meiosis in males and render *obp* genes subject to dosage compensation in females by inactivation of one X-chromosome. However, if they stay close to the pseudoautosomal boundary (as is the case in horse), they may escape X-inactivation, leading to sex-specific differences in expression [100]. This observation been made for human X-specific genes like *nlgn4x* [101], which in most mammals flanks the *obp* genes (Fig. 2). More profoundly, a similar relocation of genes from the PAR to the Y-specific region would effectively create male-specific gene copies. In the case of *obp*, such relocation could lead to the origin of male-specific pheromone-binding proteins and thus a sexually dimorphic chemical signal. In pig, such relocation may have already happened, as a single *obp* gene copy was found at 8.73 Mb from the end of the Y-chromosome, outside the PAR (Fig. 2). In the expanding field of mammalian genomics, sequencing of the Y-chromosome has lagged behind due to sequencing difficulties caused by the high abundance of repeat segments [65]. Targeted sequencing efforts focusing on Y-chromosomes may reveal additional examples of Y-specific *obp* genes.

The history of the *obp* repertoire in mammals as reconstructed here matches a birth-and-death model of gene family evolution [102] with frequent gene duplication and loss. This process has created substantial variation in *obp* gene repertoires across placental mammals. The loss of *obp* genes in several lineages could either indicate a reduced importance of chemical communication or the functional replacement of *obp* by other proteins. The apparent pseudogenization of *obp* genes in pinnipeds and cetaceans could be linked to their shift to a marine life where chemical communication may be replaced by acoustic and tactile signals. In contrast, the presence of expanded repertories in canids, horse, horned mammals and rodents could be interpreted as reflecting an increased importance of OBP in chemical communication. Yet, this hypothesis is difficult to substantiate: the dingo for example has only two *obp* genes but there is no indication that scent communication has become less important in these feral dogs compared to their domesticated relatives. A large repertoire of OBP proteins may not even be required for effective chemical communication. Indeed, a small number of ligand-specific OBP may suffice if only a limited number of ligands is involved in chemical communication. In addition, some proteins may be capable of binding a multitude of ligands, as has been demonstrated for aphrodisin in the golden hamster (*Mesocricetus auratus*) [59]. Finally, a single gene may suffice to produce multiple odorant-binding isoforms. In pig, proteins encoded by the same lipocalin genes can undergo different posttranslational modifications, creating isoforms with different ligand affinities [92, 103].

Given the multitude of secretions and tissues in which various OBP proteins have been found across species, gene diversification may have been paralleled by an equally dynamic evolution of gene expression. Expression variation that includes both signal sending and receiving secretions/tissues can even be found among closely related genes within a single species, as evidenced by OBP in the mouse (Fig. 4) [89]. Use of the same proteins at signaling and perceiving ends might facilitate the adaptive evolution of chemical communication, as it does not require coevolution of different genes or protein families on both sides. Instead, mutation of a single protein family may allow for simultaneous adaptation of both processes. With the current knowledge of OBP expression, it is too early to reliably infer the timing and order in which new expression sites emerged during mammalian evolution and the reconstruction in Fig. 4 should be interpreted very cautiously. However, OBP expression in anal sac glands very likely originated in an ancestral carnivore after its divergence from pangolins and before the divergence of dogs and foxes, since (1) these glands do not exist in non-carnivores and (2), the three OBP isoforms found here duplicated before the dog-fox divergence. In contrast, expression of *obp* genes in nasal mucosa may have evolved once in an early placental ancestor (as shown in Fig. 4) or may have originated in parallel in in several taxa. Detailed comparative analyses of multiple tissues across a wide range of mammals will be required to further resolve the evolution of expression sites and related functions.

## Conclusions

For decades, carnivoran anal sacs have been mostly considered as fermentation chambers from which volatile molecules produced by symbiotic bacteria are secreted as scent signals to communicate with conspecific animals. For the first time, we identified endogenously produced proteins as an abundant component of canine anal sac glands, which are likely to play an important role in chemical communication, probably by enabling efficient transfer of volatile signals or possibly by being part of the signal themselves. These odorant binding proteins are encoded by a gene cluster that originated on the X-chromosome early in placental mammal evolution and maintained a role in chemical communication across mammalian taxa by shifting expression to various tissues involved in both signalling and receiving scent molecules.

## Methods

### Animals

Anal sac secretions were obtained from six male and 11 female dogs of different breeds and age categories (Additional file 1). Four of the female dogs were sampled two times, once in oestrus and once in anoestrus. Oestrus samples were taken when male housemates mounted the female, or around the time that blood progesterone levels reached 2 ng/ml (in dogs where blood was taken for breeding purposes).

### Protein analysis

#### Collection of anal sac gland secretion

Dogs were handled by their owners while one of the two anal sacs was emptied by gently squeezing the surrounding tissue. Secretion was collected in a 10 ml falcon tube, and immediately transported on ice to the lab. Samples were vortexed for 30 s and centrifuged for 15 min at 21,000*g* at 4 °C. Twenty microliter of supernatant was detained for total protein concentration measurement by using the Pierce BCA Protein Assay Kit (Sigma-Aldrich). The remaining supernatant was divided into aliquots and stored at − 80 °C until further handling.

### RP-HPLC/MS and Edman sequencing

To conduct reversed-phase-high-performance liquid chromatography (RP-HPLC) of OBP proteins, 100 µl supernatant per sample was thawed on ice, diluted 20× in 2% (v/v) acetonitrile (ACN) − 0.05% (v/v) trifluoroacetic acid (TFA) solution (4 °C), and loaded on an activated reversed-phase adsorbent cartridge RP-C8 (Sep-Pak plus cartridge, Waters) to prepare the samples for further protein analysis. Cartridges were washed 3 times by applying 10 ml of 20% (v/v) ACN in 0.05% (v/v) TFA. Molecules were eluted with 6 ml 70% (v/v) ACN in 0.05% (v/v) TFA, and ACN was removed by 90 min of lyophilization (Speedvac SCV-100H, Savant instruments, Farmingdale, NY). The remaining volume was filled up to 2 ml with 1% (v/v) ACN in 0.1% (v/v) TFA and loaded onto a 150 × 4.6 mm, 5 µm Proto300 C4 HPLC column (Higgins Analytical Inc., Mountain View, CA). Proteins were eluted using a gradient of ACN in 0.1% (v/v) TFA at 1 ml/min, increasing from 0 to 80% (v/v) ACN in 80 min. Absorbance was measured at a wavelength of 214 nm. Mass spectra of all fractions were measured in parallel on an AmaZon SL ion trap mass spectrometer (Bruker Daltonics, Bremen, Germany). To visualize the protein content of both unfractionated samples and HPLC peak fractions, aliquots were adjusted to 10 mM TRIS, loaded on SDS-PAGE precast gels (Any kD Mini-PROTEAN TGX, Bio-Rad), and silver-stained (Silverquest Silver Staining kit, Invitrogen). Protein gel bands were transferred onto a polyvinylidene difluoride membrane by semi-dry blotting (Trans Blot Turbo System, Bio-Rad) and stained with 0.1% Coomassie brilliant blue R-250 (Sigma-Aldrich). Bands were excised and destained with methanol, for N-terminal amino-acid sequencing using Edman degradation (491 Procise cLC protein sequencer, Applied Biosystems).

Proteins in 100 µl aliquots of OBP peak fractions were concentrated to 50 μl and 5 μl was used for analysis with nano-scale RP-HPLC (Ultimate 3000 RSLCnano system, Thermo Scientific). Purification of proteins was accomplished using a 5 × 0.3 mm PepMap 300 C4 pre-column (Thermo Scientific) combined with a 50 × 0.15 mm Proto 300 C4 column (Higgins Analytical Inc.). Samples were loaded in 4% (v/v) ACN in 0.1% (v/v) TFA and elution was performed with an ACN gradient in 0.08% (v/v) formic acid (flow rate of 0.5 μl/min). The column effluent was directly injected into an AmaZon speed electron transfer dissociation (ETD) ion trap mass spectrometer (Bruker Daltonics) with target mass at 2200 m/z. Averaged profile spectra of proteins were obtained using Bruker Daltonics deconvolution software (data analysis 4.1). The experimentally obtained relative molecular weight (Mr) of the proteins was compared to the theoretical Mr. To calculate the theoretical Mrs, predicted RNA precursor sequences were obtained from the dog genome (CanFam 3.1, NCBI Genome Browser), manually adjusted if needed, and translated into proteins using the ExPASy Translate tool [104]. Signal Peptides were predicted using SignalP 5.0 [105] and determined by peptide abundance data inferred from our LC–MS/MS analyses, and removed before the theoretical Mr of mature proteins were calculated on the Genscript website [106].

### Tandem mass spectrometry

Forty microliter supernatant per sample was thawed on ice, diluted 20×, and subjected to RP-C8 cartridge protein purification as described above, but in this case cartridges were washed with a 2% ACN–0.05% TFA solution, molecules were eluted with 90% (v/v) ACN in 0.05% (v/v) TFA, and lyophilisation lasted for 1–5 h. Lyophilized RP-C8 eluates were dissolved in lysis buffer (8 M urea, 5 mM DTT, 30 mM TRIS). Twenty microgram of proteins were reduced and alkylated, diluted in 50 mM ammonium bicarbonate (Fluka) to 2 M of urea and digested overnight at 37 °C with 0.2 μg of trypsin (Pierce MS grade, Thermo Scientific). The peptide samples were desalted using Pierce C18 spin columns (Thermo Scientific) according to the manufacturer's instructions. Samples (0.5 μg/5 μl) were separated in an Ultimate 3000 (Thermo Scientific) UPLC system, followed by a Q Exactive Orbitrap mass spectrometer (Thermo Scientific). The Ultimate 3000 UPLC system (Dionex, Thermo Scientific) was equipped with an Acclaim PepMap100 pre-column (C18 particle size 3 μm pore size–100 Å, diameter 0.075 mm, length 20 mm, Thermo Scientific) and a C18 PepMap RSLC analytical column (particle size 2 μm, pore size–100 Å, diameter 50 μm, length-150 mm, Thermo Scientific) using a linear gradient (0.300 μl/min). The composition of buffer A is pure water containing 0.1% formic acid. The composition of buffer B is pure water containing 0.08% formic acid and 80% acetonitrile. The fraction of buffer B increased from 0–4% in 3 min, from 4–10% in 12 min, from 10–35% in 20 min, from 35–65% in 5 min, from 65–95% in 1 min, stayed at 95% for 10 min. The fraction of buffer B decreased from 95–5% in 1 min and stayed at 5% for 10 min. The Q Exactive Orbitrap mass spectrometer (ThermoScientific) was operated in positive ion mode with a nanospray voltage of 2.1 kV and a source temperature of 250 °C. Pierce LTQ Velos ESI positive ion calibration mix (Thermo Scientific) was used as an external calibrant. The instrument was operated in data-dependent acquisition mode with a survey MS scan at a resolution of 70,000 (fwhm at m/z 200) for the mass range of m/z 400–1600 for precursor ions, followed by MS/MS scans of the top ten most intense peaks with + 2, + 3, + 4, and + 5 charged ions above a threshold ion count of 1e+6 at 17,500 resolution using normalized collision energy of 25 eV with an isolation window of 3.0 m/z, apex trigger of 5–15 s and dynamic exclusion of 10 s. All data were acquired with Xcalibur 3.1.66.10 software (ThermoScientific). For protein identification, we used MASCOT version 2.2.06 (Matrix Science) against Uniprot *Canis lupus familiaris* protein databases to which we added all possible exon splice variants of the three *obp* genes, yielding 29,672 sequences. The parameters used to search at MASCOT were: parent tolerance of 10 PPM, fragment tolerance of 20 mmu, variable modification deamidation of NQ and oxidation of M, fixed modification with carbamidomethyl C and up to two missed cleavages for trypsin. To calculate the FDR [107] and judge the protein inference, the sample-specific mgf files were loaded into Scaffold 3.6.5. The proteins were quantified in Progenesis version 4.0 (Nonlinear dynamics) based on the normalized abundance of all matching features. All compound ion abundances y_i_ have been multiplied by a scalar factor α_k_ to give a normalised abundance

$${\text{y}}^{\prime }_{{\text{i}}} :{\text{y}}^{\prime }_{i} = \alpha_{{\text{k}}} {\text{y}}_{{\text{i}}} .$$

To implement the normalisation, the most suitable reference sample was determined and the scalar factor ratio between each sample being normalised and the reference sample was calculated.

### Genome screening for obp genes

A total of 98 mammalian genomes was screened to investigate the diversity and organisation of *obp* genes and retrieve their sequences for phylogenetic analyses. We used two screening strategies. First, a homology-based strategy involved genome-wide BLAT, BLASTn and tBLASTn searches against the UCSC genome browser Databases, the NCBI Genome Data Viewer, and Ensembl Genome Browser using previously retrieved *obp* transcripts and proteins as query sequences. Second, a synteny-based strategy involved detailed screening of intergenic regions between genes that flank *obp* genes previously identified in other genomes. At first instance, our screening effort focused on the genomes of three dog breeds and dingo as well as genomes representing major mammalian clades. Eventually, 102 *obp* genes were retrieved from 40 genomes (Additional file 4) to compile a data set for phylogenetic analyses (see below). If no *obp* homologues were found in the selected representative of a specific clade, additional genomes of the same clade were screened to confirm the absence of *obp* genes in the entire clade. This approach resulted in the screening of 19 bat genomes, 29 primate genomes, two monotreme genomes and five marsupial genomes.

### Phylogenetic reconstruction of obp gene evolution

The coding sequences of the 102 retrieved *obp* genes (Additional file 4) were aligned using the E-INS-I algorithm with default parameters implemented in Mafft v7 [108]. A general time-reversible model with a gamma distribution to accommodate among-site rate heterogeneity and an estimated proportion of invariable sites (GTR + G + I) was identified as the best-fitting DNA substitution model using Akaike and Bayesian information criteria in ModelTest-NG 0.1.6 [109]. Consequently, this model was applied for Bayesian phylogeny inference with using MrBayes 3.2.7a [110] and maximum likelihood bootstrap analyses, sing RAXML 8.2.12 [111], both accessed through the CIPRES Science Gateway [112]. For the Bayesian analyses, two runs of four Markov chain Monte Carlo chains each were run in parallel for 12 million generations, with a sampling interval of 1000 generations and a burnin of two million generations. Convergence of the parallel runs was verified by split frequency standard deviations (< 0.01) and potential scale reduction factors (approximating 1.0) for all model parameters. Maximum Likelihood bootstrap support values were obtained by performing 1000 “rapid” bootstrap replicates in RAXML. The consensus trees resulting from the Bayesian and bootstrapping analyses were nearly identical in topology and the few conflicting branches received weak support by either analysis. In the absence of closely related outgroup sequences, phylogenetic analyses were performed on ingroup (*obp*) sequences only, producing unrooted gene trees.

The resulting Bayesian consensus phylogram was used to reconstruct gene duplication events and gene losses on the mammalian taxon tree using a gene tree/species tree reconciliation (GTSTR) analysis under the parsimony principle as implemented in Notung 2.9 [113]. A taxon tree that matches our gene tree was retrieved from the timetree.org database [84] as a chronogram with divergence times as averages calculated from a database of published molecular clock estimates. The cost of gene duplications and losses were both set to 1.0. We made two reconstructions that represent contrasting prioritisation strategies. A first GTSTR was based on the unchanged Bayesian gene tree without allowing rearrangements of weakly supported branches even if this would improve the reconciliation (yield lower estimated number of duplications and losses). This reconstruction prioritises confidence in the gene tree over optimizing tree reconciliation. A second GTSTR allowed the rearrangement of weakly supported branches if this results in lower estimated number of duplications and losses. This reconstruction prioritises minimization of the number of events over maintaining unreliable branches. Branches were considered weakly supported if they received Bayesian posterior probabilities < 0.9 and bootstrap percentages < 70%. As expected, application of the unchanged tree implicated a higher number of gene duplications and losses than a partially rearranged tree (Additional file 6). Both reconstructions also allowed us to tentatively root the gene tree, by defining the branch on which the root position would implicate the least number of gene duplication events and losses. Under both reconstruction strategies, the most optimal root was located on the branch separating the xenarthran and afrotherian sequences from all other sequences (Additional file 5).

In the current absence of comprehensive expression information for the majority of species, the evolution of *OBP* secretion sites was reconstructed on a pruned tree including the 13 genes for which expression information could be inferred from the present study or previous proteomic studies (Additional file 7). The origin of expression sites was mapped on this gene tree using the maximum parsimony principle as implemented in Mesquite 3.61 [114].

## Supplementary Information

**Additional file 1.** Information on individual dogs and total protein concentrations of ASGS samples.

**Additional file 2. **Normalized abundances of OBP isoform in dog ASGS of pure breeds, crossbreeds, and mixed breeds. Although OBP abundances are higher in mixed breeds, more samples of different breeds are required to validate this pattern.

**Additional file 3. **Proteins identified by LC-MS/MS in the anal sac gland secretions of 21 samples (17 dogs). All 56 proteins are listed in major functional classes. For each protein, domain families, Uniprot accession numbers and normalised abundance per sample are provided.

**Additional file 4. **List of identified *obp* genes per species, their accession numbers, and the genomes from which they were retrieved. All of these genes were included in the phylogenetic analyses.

**Additional file 5. **Bayesian consensus phylogram for 102 *obp* genes, retrieved from 40 placental mammal genomes. Numbers near branches indicate Bayesian posterior probabilities (BPP; left) and nonparametric bootstrap percentages (NPBS; right). Branches supported by BBP > 0.95 and NPBS > 75% are drawn as thick lines. Branches supported by BBP < 0.90 and NPBS < 70% (indicated in red) are drawn as dashed lines and were allowed to be rearranged in a gene tree/species tree reconciliation analysis (see Fig. 3). Branch colours delineate *obp* sequence clades unique to one of the following mammalian taxa: Carnivora (yellow), Pecora (light blue), Cetacea (teal), Pig (pink), Perissodactyla (light brown), Pholidota (green), Rodentia (orange) and Eulipotyphla (dark brown), Afrotheria (light green), Xenarthra (grey).

**Additional file 6. **Alternative reconstruction of the evolutionary history of the *obp* gene repertoire in placental mammals. Similar to Fig. 3, *obp* gene lineages are drawn as thin branches superimposed on a timetree of placental mammals, with gene duplication events and gene losses along branches depicted as blue vertical bars (I) and red crosses (X), respectively. Ancestral and extant gene repertoires are schematically shown at key internal nodes and terminal nodes, respectively. This reconstruction was obtained by gene tree/species tree reconciliation with the consensus phylogram from the Bayesian analyses (Additional file 5) as a fixed gene tree. Unlike the reconstruction in Fig. 3, arrangement of weakly supported branches to minimise the inferred number of gene duplication events and losses was not allowed prioritising confidence in the inferred gene tree of maximising the reconciliation. As a result, this reconstruction implicates substantially more duplication events (indicated by light blue vertical bars), losses (indicated by light red crosses) and ancestral genes for basal branches (coloured light blue).

**Additional file 7. **Overview of *obp* genes with previously identified secretion sites of their corresponding proteins.

## Data Availability

All protein and DNA sequences analysed in this study are available in the NCBI and Ensembl databases and can be accessed using the accession numbers provided in Additional file 3. A fasta files of the DNA sequences and HPLC chromatograms are available from the corresponding author upon reasonable request.

## Abbreviations

ASGS: Anal sac gland secretions
GTSTR: Gene tree/species tree reconstruction
OBP: Odorant binding protein
PAR: Pseudoautosomal region
