# Supplementary material for: Odorant-binding proteins in canine anal sac glands indicate an evolutionarily conserved role in mammalian chemical communication
Source: BMC Ecol Evol. 2021 Sep 26;21:182. doi: 10.1186/s12862-021-01910-w (PMC8474896; doi:10.1186/s12862-021-01910-w)
Supplement: Supplementary file 1 — Additional file 1. Information on individual dogs and total protein concentrations of ASGS samples. [file 12862_2021_1910_MOESM1_ESM.pdf]

Additional file 1: Information on individual dogs, and total protein concentrations of anal sac gland secretion samples

	Information of individual dogs							Anal gland samples
**nr**	**age at sampling**	**age group**	**gender**	**breed**	**inbreed**	**origin**	**born**	**protein (mg/ml)**
**1**	6m	YOUNG	male	great dane	PUREBRED	Belgium	home	**733,94**
**2**	7m	YOUNG	male	labrador	PUREBRED	Belgium	home	**419,386**
**3**	1y	YOUNG	male	mix (medium dog)	MIXED BREED	Romania	street	**209,431**
**4**	4y	ADULT	male	mix (shepherd-like)	MIXED BREED	Spain	street	**219,847**
**5**	11y	OLD	male	labrador x golden retriever x malinois	CROSSBREED	Belgium	home	**37,807**
**6**	14y	OLD	male	mix (shepherd-like)	MIXED BREED	Greece	street	/
**7**	5m	YOUNG	female	Mix (husky/podenco-like)	MIXED BREED	Spain	street	**280,107**
**8**	5m	YOUNG	female	great dane	PUREBRED	England	home	**264,119**
**9**	6m	YOUNG	female	great dane	PUREBRED	Belgium	home	**420,271**
**10**	2y	YOUNG	female	great dane	PUREBRED	Belgium	home	**451,372**
**11**	3y	YOUNG	female	mix (small dog)	MIXED BREED	Romania	street	**868,677**
**12**	4y	ADULT	female	mix (podenco-like)	MIXED BREED	Spain	street	**198,139**
**13**	4y	ADULT	female	great dane	PUREBRED	Belgium	home	**13**
**14**	7y	ADULT	female	great dane	PUREBRED	Belgium	home	**739,517**
**15**	10y	OLD	female	labrador x german shepherd	CROSSBREED	Belgium	home	**580,196**
**16**	12y	OLD	female	labrador x border collie x boxer	CROSSBREED	Belgium	home	**404,382**
**17**	14y	OLD	female	border collie x labrador	CROSSBREED	Switzerland	home	**6,042**
**7 (oestrus)**	2y							**594,94**
**9 (oestrus)**	11m							**550,811**
**10 (oestrus)**	2y							**141,77**
**13 (oestrus)**	4y							**5,441**
