# Supplementary material for: Odorant-binding proteins in canine anal sac glands indicate an evolutionarily conserved role in mammalian chemical communication
Source: BMC Ecol Evol. 2021 Sep 26;21:182. doi: 10.1186/s12862-021-01910-w (PMC8474896; doi:10.1186/s12862-021-01910-w)
Supplement: Supplementary file 7 — Additional file 7. Overview of obp genes with previously identified secretion sites of their corresponding proteins. [file 12862_2021_1910_MOESM7_ESM.pdf]

Additional file 7: *obp* genes and secretion sites of their corresponding proteins.

**Gene name**	**Secreted protein**	**Reference**
dog *obp3*	skin dander	Mattsson et al. 2010
cattle *obp3*	nasal mucosa	Bignetti et al. 1985
cattle *obp9*	skin dander	Rautiainen et al. 1998
pig *obp4*	nasal mucosa	Paolini et al. 1998
elephant *obp2*	nasal mucosa	Lazar et al. 2002
mouse *obp1*	vaginal secretion	Stopková et al. 2014; Černá et al. 2017
mouse *obp2*	vaginal secretion	Stopková et al. 2014; Černá et al. 2017
mouse *obp5*	vaginal secretion, saliva, tears, nasal mucosa	Pes et al. 1998; Stopková et al. 2014, 2017; Stopka et al, 2016; Černá et al. 2017
mouse *obp6*	vaginal secretion, saliva, tears, nasal mucosa	Stopková et al. 2014, 2017; Stopka et al, 2016; Černá et al. 2017; Kuntová et al. 2018
mouse *obp7*	vaginal secretion, saliva, tears, nasal mucosa	Pes et al. 1998; Stopková et al. 2014, 2017; Stopka et al, 2016; Černá et al. 2017
mouse *obp8*	vaginal secretion, tears	Stopková et al. 2014, 2017; Černá et al. 2017
hamster *msp*	saliva, tears	Thavathiru et al. 1999
hamster *flp*	tears	Ranganathan et al. 1999; Srikantan et al. 2005
hamster *aphrodisin*	vaginal secretion	Briand et al. 2004a
